# Efficacy of *Hovenia dulcis* Fruit Extract in Hangover Mitigation: Double-Blind Randomized Clinical Evaluation

**DOI:** 10.3390/foods13244084

**Published:** 2024-12-17

**Authors:** Dong Hyun Paik, Ki Won Lee, Youn Young Shim, Martin J. T. Reaney, Ilbum Park, Sang-Hun Lee, Jong-Yul Park, Euddeum Park, Sung-Bum Lee, In Ah Kim, Guangpeng Xu, Ji Youn Hong, Young Jun Kim

**Affiliations:** 1 Natural Products Convergence R&D Division, Kwangdong Pharmaceutical Co., Ltd., Gwacheon 13840, Republic of Koreapib975@ekdp.com (I.P.); spelljjt@ekdp.com (S.-H.L.); 11075@ekdp.com (J.-Y.P.); 13130@ekdp.com (E.P.); 2 Department of Food and Bioproduct Sciences, University of Saskatchewan, Saskatoon, SK S7N 5A8, Canada; martin.reaney@usask.ca; 3 Prairie Tide Diversified Inc., Saskatoon, SK S7J 0R1, Canada; 4 Department of Family Medicine, Soonchunhyang University Bucheon Hospital, Bucheon 22972, Republic of Korea; 5 Global Medical Research Center, Seoul 06526, Republic of Korea; 6 Department of Food and Biotechnology, Korea University, Sejong 30019, Republic of Korea; xuguangpeng@korea.ac.kr; 7 Department of Food Regulatory Science, Korea University, Sejong 30019, Republic of Korea; khjy1025@korea.ac.kr

**Keywords:** alcohol, *Hovenia dulcis* Thunb., hangover, acute hangover scale, alcohol metabolism, clinical trials

## Abstract

The fruit of *Hovenia dulcis* Thunb. (HD) is renowned for its medicinal properties and is rich in bioactive compounds, traditionally used in East Asian medicine as a natural antidote for alcohol intoxication. A randomized, double-blind, crossover, placebo (PLA)-controlled clinical trial was conducted to compare the effects of beverages containing 0.475% HD (HDB), HD combined with 0.1% *Pueraria lobata* extract (HDPB), and HD combined with 0.02% glutathione yeast extract (HDGB) with the PLA on the symptoms of a hangover. Subjects (*n* = 30) were randomized into six test groups consuming three beverages, including the PLA. After ingestion, blood alcohol and blood acetaldehyde concentrations were measured at 0, 0.25, 0.5, 1, 2, 4, 6, and 15 h post-alcohol consumption. No significant differences in hematology tests and vital signs were observed amongst the treatment groups; however, at 0.5 and 6 h, the blood alcohol concentrations of the HDB and HDPB groups were significantly lower compared to the PLA group (*p* < 0.05). Additionally, the blood acetaldehyde concentrations in the HDPB group showed significantly lower values than the PLA after 6 h *(p* < 0.05). These findings suggest that HD may aid in alcohol detoxification and limit acetaldehyde accumulation. This indicates the potential for HD as a functional food ingredient for alleviating hangover symptoms.

## 1. Introduction

A hangover from alcohol is characterized by a collection of symptoms that appear the day after heavy drinking, usually when the blood alcohol level is nearly zero [1]. Physiological symptoms of hangovers include thirst, drowsiness, headache, fatigue, and sweating, while mental symptoms may involve dizziness, depression, anxiety, and irritability [2]. These symptoms not only cause discomfort but also impair cognitive and physical functions, leading to significant social and economic impacts [3]. The pathophysiology of hangovers is complex and multifactorial, involving dehydration, electrolyte imbalance, intestinal disturbances, and accumulation of toxic metabolites such as acetaldehyde [4,5]. Therefore, many hangover remedies are marketed every year but there is currently a lack of clinical trials to evaluate their solid effectiveness.

Alcohol metabolism begins primarily with its absorption and transport through the bloodstream to the liver. In the liver, alcohol is converted to acetaldehyde by alcohol dehydrogenase (ADH) [6]. Acetaldehyde is then further oxidized to acetate by aldehyde dehydrogenase (ALDH). The acetate is finally broken down into carbon dioxide and water, which are eliminated from the body [7]. Excessive alcohol consumption can lead to liver damage, typically indicated by elevated levels of liver enzymes such as alanine aminotransferase (ALT) and aspartate aminotransferase (AST) [8,9]. These enzymes are markers of liver cell injury, and their elevated levels can signal liver inflammation or damage. In addition, alcohol metabolism produces reactive oxygen species, which can cause oxidative damage to various tissues, including the liver, brain, and gastrointestinal tract, and are a significant cause of hangover symptoms.

In recent years, natural remedies for the prevention and treatment of hangovers have garnered increasing attention [10]. Compared to conventional medications—which can cause serious side effects such as ataxia, impaired attention, and confusion—natural bioactive compound products are considered safer alternatives [11]. For example, *Mesembryanthemum crystallinum* (ice plant) [12], *Pueraria lobata* (kudzu flower) [13], and *Artemisia* herbs [14], plant extracts, and glutathione yeast extract (GY) [15], which is a fermented extract, have been shown to relieve hangover symptoms or promote alcohol metabolism.

This study aims to evaluate the efficacy of *Hovenia dulcis* Thunb. extract (HD), *P. lobata* root extract (PL), and GY as natural remedies for hangover relief. HD exhibits strong antioxidant activity due to its high flavonoid content [16,17,18], lowering liver enzyme levels—including ALT and AST—improving liver function, and preventing pathological changes in liver tissue [19]. Polysaccharides derived from HD were reported to regulate hepatic fatty acid metabolism, reduce inflammation [20], and support the liver’s protection and xenobiotic metabolism [21]. HD also enhances alcohol metabolism by increasing ADH and ALDH activity [22] while reducing fatigue [23]. These activities highlight its potential as a functional food for hangover prevention.

PL exhibits strong antioxidant activity due to its polyphenols, including puerarin [24]. The antioxidant property of this substance helps to neutralize free radicals, thereby protecting cells from oxidative stress. Additionally, PL has been demonstrated to enhance blood circulation and forestall the development of cardiovascular diseases [25], which is of paramount importance for maintaining optimal heart health. It also safeguards nerves [26], which can help in preventing neurodegenerative diseases, and manages metabolic disorders [27], such as diabetes and obesity. Furthermore, it alleviates alcohol-induced liver damage [28], supporting liver health and function.

Similarly, GY has been reported to have strong antioxidant activity [29], enhancing the body’s ability to combat oxidative stress. This extract has been demonstrated to enhance the immune system [30], thereby providing a robust defense against infections and diseases. It protects nerves [31], similar to PL, which suggests a potential synergistic effect in neuroprotection. Additionally, GY prevents metabolic disorders such as diabetes [32], contributing to overall metabolic health. It also supports liver detoxification [33], aiding in the removal of toxins and improving liver function. The combined use of PL and GY leverages their shared antioxidant properties and complementary health benefits. When combined, they can provide enhanced protection against oxidative stress, support cardiovascular and liver health, improve metabolic function, and offer neuroprotective effects. This synergistic approach highlights their potential as effective natural remedies for promoting overall human health and well-being.

The objective of this study is to conduct clinical trials to evaluate the efficacy of HD, PL, and GY, both individually and in combination, in preventing hangover symptoms. By examining their potential as natural remedies, this study is designed to contribute to the development of safer and more effective treatments for hangovers, potentially alleviating their significant social and economic impacts.

## 2. Materials and Methods

### 2.1. Study Participants and Inclusion/Exclusion Criteria

The Global Medical Research Center located in Seoul (Republic of Korea) enrolled participants in this clinical trial. The inclusion criteria were based on Chang and Kim [34] but modified as follows: (1) aged between 19 and 40 years; (2) body mass index (BMI) between 18.5 and 25 kg/m^2^; (3) a history of experiencing hangovers. Subsequently, participants were required to meet the following criteria: (1) consumption of alcohol within the previous 30 days; (2) exhaled alcohol concentration of 0.00% at the initial visit on Day 2; and (3) consent to participate in a human clinical trial, along with a signed consent form. The criterion related to the experience of hangovers meant that participants must have previously experienced hangover symptoms, such as headaches, nausea, fatigue, and sensitivity to light and sound following alcohol consumption. This ensured that the study population was relevant to the research on hangover alleviation.

Individuals who did not meet the following criteria were excluded from participation: (1) individuals undergoing treatment for a serious medical condition, including but not limited to cardiovascular (hypertension), immune, respiratory, endocrine (diabetes), renal and urinary, neurologic and musculoskeletal, psychiatric, infectious disease, and malignancy; (2) individuals with a history of peptic ulcer disease, gastroesophageal reflux disease, or a history of serious gastrointestinal disease (e.g., Crohn’s disease); (3) individuals who are pregnant or lactating or who intend to become pregnant during the course of this human application study; (4) individuals with an alcohol use disorder or recent heavy alcohol consumption that may affect the study results; (5) individuals taking medications that affect alcohol metabolism (e.g., antidepressants) or pose the risk of gastrointestinal bleeding (e.g., warfarin, clopidogrel, aspirin, non-steroidal anti-inflammatory drugs); (6) individuals who have used medications that enhance liver function (disulfiram class); (7) individuals who have used drugs, dietary supplements, or medications that affect drug-metabolizing enzymes within a specified time period prior to the screening visit; (8) individuals with abnormal laboratory results (e.g., AST, ALT, GPT), including creatinine, thyroid-stimulating hormone, fasting blood glucose levels; (9) individuals who have participated in another interventional clinical trial within the past month or intend to participate in another clinical trial during this study; (10) individuals with known sensitivities or allergies to any of the food trial ingredients; and (11) other reasons for exclusion at the discretion of the investigator.

### 2.2. Study Design

**Figure 1:**
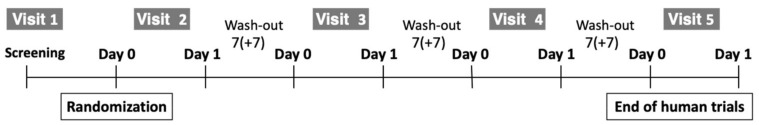
Overview of human clinical trial.

This study was approved by the Institutional Review Board of the Global Medical Research Center (IRB No. GIRB-24216-ZL) and registered with the Centers for Disease Control and Prevention Clinical Research Information Service (CRIS No. KCT0009899). It was conducted from April 29 to June 16, 2024. This study was designed as a randomized, double-blind, parallel-group, placebo (PLA)-controlled trial (Figure 1). On the day of screening, participants were randomly assigned to different groups, including one PLA group and three test groups, using a double-blind method. A statistician prepared the randomization list in collaboration with the sponsor. Both participants and researchers were unaware of group allocation until the end of the study. Power analyses were performed to estimate the sample size required to test for superiority [35].

The human clinical trial was a crossover design trial with randomization to one of six arms. The required number of human clinical trial subjects was 30 to account for the dropout rate (20%) (Table 1). Participants were sent for the human clinical trial at Visits 1 and 2 (Day 0). Participants were screened to determine if they met the inclusion/exclusion criteria by completing questionnaires on demographics, lifestyle, medical history, and medication history. They also underwent physical examination, vital signs (blood pressure, pulse), anthropometry (height, BMI, weight), clinical pathology tests, and pregnancy tests (for women of childbearing age) [36]. The drinking behavior questionnaire included questions about the type of alcohol, amount consumed, number of times per week, and hangovers experienced in the past month. At Visit 2 (Day 0), all human subjects meeting the inclusion/exclusion criteria were assigned to each of the following arms by assignment code using blocked randomization [37]. To ensure balanced randomization between the intake arms, the ratio of the number of human subjects in each arm was equalized to 1:1:1:1:1:1. Randomized human subjects received a single, alternating dose of the investigational food (Test Food I or Test Food II or Test Food III or control food) at Visits 2, 3, 4, and 5, respectively, with a 7-day washout period between each pair of consecutive visits (Figure 1).

### 2.3. Interventions

The concentrated aqueous samples were kindly provided by Kwangdong Pharmaceutical Co., Ltd. (Gwacheon, Republic of Korea). Four samples, including the control (PLA), were administered orally as 500 mL beverages, the specific compositions of which are shown in Table 2: 0.475% (*w*/*v*) HD beverage (HDB); HD combined with 0.1% (*w*/*v*) PL (HDPB); and HD combined with 0.02% GY (HDGB). The PLA sample consisted of 0.012% caramel color powder, and 0.070% flavor dissolved in purified water. In addition, three samples contained vitamin C (0.020%), sodium bicarbonate (0.023%), glycine (0.010%), and flavorings (0.105%) as minor ingredients, all ingredients are expressed in weight per volume. Each sample was identical in appearance, shape, color, taste, sweetness, packaging, and additives. All participants received an identical meal before the test and consumed either the PLA or the intervention beverage 2 h after the meal. Thirty minutes after ingesting the test substance, the participants consumed whiskey (40% alcohol, 0.9 g/kg body weight) in two 30-minute increments. To control for variables and ensure the accuracy and reliability of experimental results, subjects were instructed to fast after alcohol ingestion. At each visit, subjects were instructed to abstain from alcohol for 24 h prior to the next visit. Upon waking the next morning, subjects completed a hangover symptom questionnaire 15 h after alcohol ingestion.

### 2.4. Outcome Measures

#### 2.4.1. Acute Hangover Scale (AHS)

The AHS is used to assess hangover symptoms and their severity [38]. This scale consists of 9 items that rate the severity of each symptom and provide an overall hangover severity score. Each item is scored from 0 to 7, with the total AHS score being the average of all items. The symptom severity is categorized as ‘none’ (0 points), ‘mild’ (1 point), ‘moderate’ (4 points), or ‘incapacitating’ (7 points).

#### 2.4.2. Alcohol and Acetaldehyde Analysis During Blood Sample Handling and Collection

Blood was taken at 0, 0.25, 0.5, 1, 2, 4, 6, and 15 h after alcohol consumption at Visits 2, 3, 4, and 5 (Days 0 and 1). A catheter filled with saline for the injection was placed in a vein in the participant’s arm to obtain 5 mL of blood at each time point. Blood samples were placed in BD Vacutainer^®^ NaF tubes (BD, Milan, Italy), stored at 4 °C, and analyzed immediately according to the guidelines of the external laboratory [39]. Samples were discarded immediately after analysis and not used for any other purpose.

#### 2.4.3. Analysis of Alcohol and Acetaldehyde in Blood

All clinical blood samples were stored as described in Section 2.4.2 and processed for ethanol and acetaldehyde concentration analyses. Blood samples treated with anticoagulants were centrifuged, and the supernatant plasma was collected. For analysis, 200 µL of human plasma was mixed with 500 µL of saturated NaCl solution in a headspace vial and 100 µL of 0.005% *n*-butanol was added. Samples were analyzed on an Agilent 5977 series GC system (Agilent Technologies Inc., Palo Alto, CA, USA) with a CTC headspace GC/MS detector (CTC Analytics AG, Zwingen, Switzerland). Ethanol, acetaldehyde, and *n*-butanol were chromatographically separated on a Discovery HP-INNOWAX column (0.32 mm × 30 m, 0.5 µm, Sigma-Aldrich, St. Louis, MO, USA). Helium was used as the carrier gas at a constant flow at 3 mL/min and the interfacial temperature was set at 200 °C. An electron ionization system with 70 eV ionization energy was used for GC-MS detection. The initial temperature of the GC oven was maintained at 35 °C for 3 min, after which it was increased to 85 °C at a rate of 40 °C per minute and held for a further 2 min. The equilibration temperature and time for the headspace sampler were 70 °C and 10 min, respectively. The injection volume was 250 µL, and the split ratio was 100:1. The mass spectrometer was operated in single-ion monitoring mode with ethanol set to *m*/*z* 45, 46, and 31 and acetaldehyde set to *m*/*z* 43, 41, and 29. Quantification was based on *m*/*z* values of 45 for ethanol and 43 for acetaldehyde [11].

### 2.5. Safety Assessments

Vital signs, including systolic and diastolic blood pressure, temperature, pulse rate, and clinical laboratory tests (hematology, biochemistry, and urinalysis) were performed on participants at screening (Visit 1) and Visit 2 (Day 1) [40]. In addition, adverse events were identified during the study through interviews or questionnaires administered to the participants.

### 2.6. ADH and ALDH Enzyme Activity Assay

To measure markers of alcohol hangover, ADH and ALDH activity were determined in plasma after blood separation. The activities of ADH (K787; BioVision Inc., Milpitas, CA, USA) and ALDH (K731; BioVision Inc., Milpitas, CA, USA) were determined using commercial kits. All assay procedures were performed according to the manufacturer’s instructions [11].

### 2.7. Statistical Analysis

Statistical analyses were performed using SAS version 9.4 from the SAS Institute (Cary, NC, USA). Data were analyzed by calculating the mean ± standard deviation (SD) with appropriate descriptive statistics, and the significance of differences was determined by two-tailed *t*-tests at * *p* < 0.05 and ** *p* < 0.01.

#### 2.7.1. Validation Variables

The degree of change in blood alcohol and acetaldehyde concentrations (hourly concentrations, C_max_, T_max_, and AUC) of each group were analyzed using repeated measures analysis of variance (RM ANOVA) [41]. ANOVA was performed to evaluate whether there was a statistically significant difference in the intake effect between each group under the assumption of no residual effect. RM ANOVA was used to analyze the difference between the intake effects of each group at each time of AHS to evaluate whether there was a statistically significant difference.

#### 2.7.2. Safety Evaluation Variables

All adverse events that occurred after the ingestion of the study food were tabulated and evaluated through the calculation of an incidence rate. The proportion of human subjects with adverse events in each group was calculated and compared using the Chi-square test or Fisher’s exact test [42]. The hematological and blood chemistry test values between the two groups (Test Food I and control food, Test Food II and control food, Test Food III and control food) were evaluated for statistically significant differences by ANOVA or Kruskal–Wallis test according to normality, and post hoc tests were performed if necessary. The frequency and proportion of normal and abnormal urinalysis were calculated and compared using the Chi-square test or Fisher’s exact test. Intra-group comparisons of pre- and post-consumption changes in vital signs (blood pressure, pulse) were analyzed using paired *t*-tests. The magnitude of change between groups (Test Food I and control food, Test Food II and control food, Test Food III and control food) was assessed for statistically significant differences using ANOVA or Kruskal–Wallis test depending on normality, and post hoc tests were performed if necessary.

## 3. Results and Discussion

### 3.1. Enrollment

**Figure 2:**
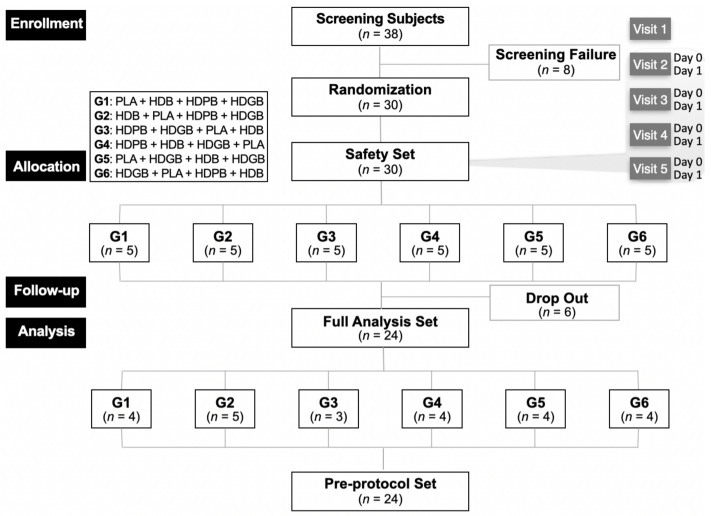
A flow chart illustrating the selection and allocation of participants in this study. Thirty eligible subjects were randomized to one of the six groups, followed by a one-week washout period, and then crossed over to the other group. All subjects completed the study.

A total of 30 participants were enrolled, with 8 excluded during the screening process, resulting in 24 participants included in the experimental analysis from 29 April 2024 to 16 June 2024. During the experimental period, six participants were excluded from the final analysis. Regarding efficacy data, the per-protocol (PP) set analysis represents the primary analytical approach, complemented by the full analysis (FA) set analysis. The PP analysis method provides the maximum potential efficacy of a treatment under strict adherence to the protocol, making it suitable for assessing the effectiveness of the protocol itself. In contrast, the FA analysis method maximizes the reflection of the real conditions of the study population, thereby enhancing the external validity of the results [43]. For demographic data, the PP set analysis is the primary method, while for safety data, the safety set analysis is used. None of the participants in the FA set were excluded from the PP set analysis set among those in the FA set; therefore, both the FA set and the PP set were analyzed with the same number of cases (*n* = 24) (Figure 2).

### 3.2. General Participant Characteristics

Participant demographics and pre-intake characteristics are compared in Table 3. All pre-intake characteristics, including demographics, were compared between the intake groups to identify factors that differed; however, the experimental group comprised 12 (50%) males and 12 (50%) females. There was no statistically significant difference in age, with a mean age of 29.19 ± 3.86 years for the experimental group. In addition, the intake groups were matched to ensure that there were no significant differences in exercise status, smoking status and amount, sleep duration, height, and weight between the two groups, thus ensuring comparability between the groups.

### 3.3. Clinical Pathology Assessment for Hangover Relief

A total of 30 subjects were randomized into the test and control groups for a human application trial to obtain more realistic safety data and conduct a comprehensive risk assessment. The primary method used was a safety set analysis [44]. No statistically significant differences were observed between each group and the PLA group for any hematological test at 15 h post-drinking (Table 4) and no serious adverse events occurred. The hematological test of monocyte analysis showed that the HDGB group had the lowest monocyte count of 6.6 ± 1.2% (*p* = 0.2959), while the other groups, including the PLA, had similar counts of 7.1–7.3%. In the blood chemistry test, ALT analysis showed that HDB and HDPB were 19 ± 15 and 17 ± 12 U/L, respectively, compared to the lower values (both 15 ± 11 U/L) for the PLA and HDGB. However, these changes were within the normal range and were not found to be clinically significant. In addition, there was no statistically significant difference between the groups in any of the urine test parameters, including glucose, ketones, and bilirubin at 15 h after drinking. Therefore, no safety concerns were reported.

Table 5 presents the data on vital signs, including blood pressure and pulse, and body measurements such as weight. According to the research results of Seppä et al. [45] and Kawano et al. [46], vital sign changes in pulse and blood pressure after drinking increased the pulse rate immediately after drinking, and blood pressure decreased due to the acute depression effect. Blood pressure began to rise after 8 h (subacute pressure effect), and blood pressure returned to its previous level 24 h after abstinence. In this study, vital signs of blood pressure and pulse were measured 15 h after drinking and no statistically significant differences were observed between the groups in any of the measured parameters; however, after 15 h, significant differences were observed between the PLA and HDB (*p* = 0.0060) or HDPB (*p* = 0.0309) in the systolic blood pressure analysis of the change from baseline using the paired samples *t*-test. In addition, in the diastolic blood pressure analysis using the paired samples *t*-test, significant differences were observed between the PLA (*p* = 0.0462) and samples (*t*-test). However, for pulse rate, the analysis showed no statistically significant differences between the groups (*t*-test).

### 3.4. Survey of the Symptoms of a Hangover

AHS scores are utilized to assess various physical and cognitive issues, including headache, nausea, fatigue, and concentration difficulties, [47,48] which are commonly associated with the severity of hangover symptoms. In this study, AHS scores following alcohol consumption were compared between the experimental groups (HD, HDPB, and HDGB) and the control group (PLA) to determine any statistically significant differences. Table 6 presents the AHS results measured 15 h after alcohol consumption, with a total score ranging from 3.0 ± 2.7 to 3.8 ± 3.3. There were no significant differences between the experimental and control groups (PLA). The experimental group exhibited similar scores to the PLA group across various symptoms, including thirst, hangovers, headaches, dizziness/fainting, anorexia, gastrointestinal disorders, nausea, and heart palpitations. In addition, there was no significant difference in alcohol consumption patterns between different GG genotypes, which is consistent with previous research suggesting that genetic factors may not significantly influence hangover symptoms in this context [49].

Previous studies have suggested that blood ethanol levels, rather than acetaldehyde, play a critical role in hangover severity [50]; however, recent evidence suggests that both systemic alcohol consumption and acetaldehyde can lead to increased acetaldehyde accumulation in both blood and brain tissue in mice [51]. In addition, a recent clinical trial also found an association between acetaldehyde levels and hangover symptoms [52,53]. Accordingly, the present study sought to ascertain the efficacy of the experimental treatment in alleviating the symptoms of alcohol withdrawal by measuring the concentrations of blood alcohol and acetaldehyde.

The peak blood alcohol concentration (C_max_), time to peak blood concentration (T_max_), and area under the concentration–time curve (AUC) from 0 to 15 h after drinking are shown in Table 7. Compared to the PLA, HDPB sample ingestion slightly reduced blood alcohol concentrations in AUC and C_max_ but there were no statistically significant differences between groups. Blood alcohol and acetaldehyde concentrations by group after alcohol ingestion are shown in Table 7. Although the AUC levels of HDPB and HDB in blood alcohol tended to be lower than those of the PLA, these differences did not achieve statistical significance (*p* = 0.0800). Additionally, no significant differences were observed in C_max_ and T_max_ among the samples. In the analysis using the paired samples *t*-test, no significant differences were observed between the PLA and HDB or HDGB. Additionally, comparative analysis of AUC, C_max_, and T_max_ for blood acetaldehyde showed no significant differences among the test groups.

### 3.5. Changes in Blood Alcohol and Acetaldehyde Levels

**Figure 3:**
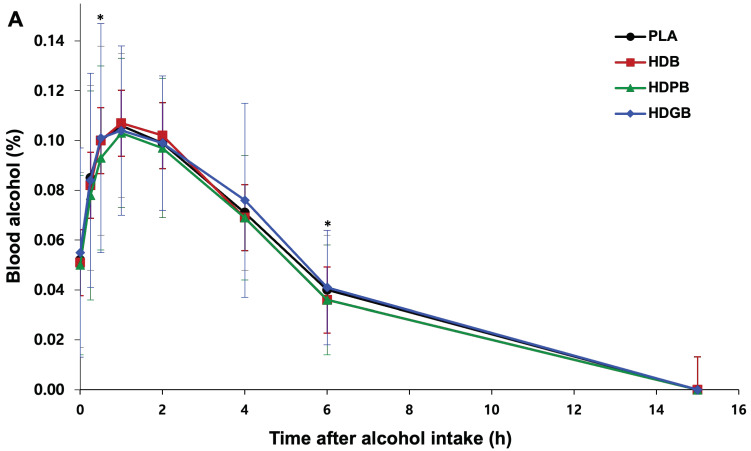

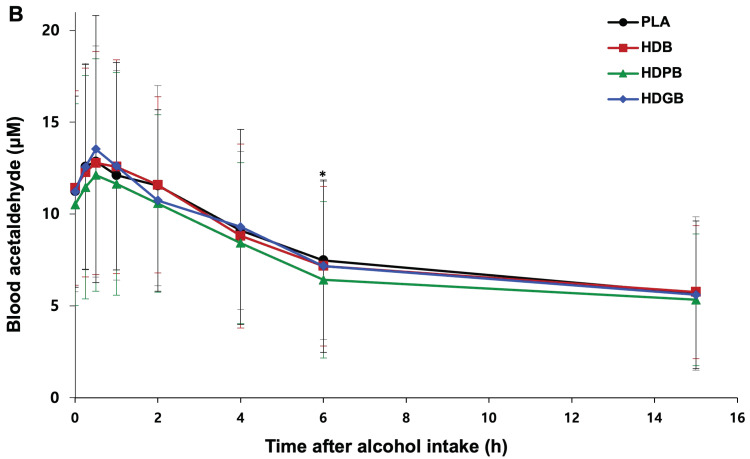
Effect of samples and PLA on (**A**) blood alcohol and (**B**) acetaldehyde concentrations after alcohol consumption at different time points (0, 0.25, 0.5, 1, 2, 4, 6, and 15 h). Group comparisons were conducted using *p*-values derived from either the two-sample *t-*test (T) or Wilcoxon rank sum test (W). * *p* < 0.05.

Figure 3 shows the changes in blood alcohol and acetaldehyde concentrations over a 15-h period following consumption. Previous research suggests that blood alcohol concentrations typically peak at approximately 1 h after drinking and then decline [54,55]. Our study also displayed this, and our comparisons between each treatment group and the PLA using the paired samples *t*-test showed no significant differences at most times (0.0, 0.25, 1.0, 2.0, 4.0, 15 h), except at 0.5 and 6 h. Interestingly, the HDPB group (0.093 ± 0.037%) had lower blood alcohol levels compared to the PLA group (0.100 ± 0.038%) at 0.5 h post-consumption (*p* = 0.0396). Although statistically significant, the observed difference is minimal and may not hold biological or practical significance in a broader context [56].

This finding is consistent with Thomes et al. [57], who observed similar trends in blood alcohol levels after alcohol consumption, suggesting that the potential of HD and PL extracts might influence alcohol metabolism. In addition, both the HDB group (0.036 ± 0.018%) and the HDPB group (0.036 ± 0.022%) had lower blood alcohol concentrations after 6 h than the PLA group (0.040 ± 0.022%) (*p* = 0.0368), further supporting the hypothesis that certain extracts may play a role in effectively modulating alcohol metabolism. In addition, the study by Park et al. [58] highlights the importance of understanding changes in blood alcohol and acetaldehyde concentrations, which are critical factors in the severity of hangover symptoms. Analysis of blood acetaldehyde concentrations showed that subjects receiving HDPB had consistently lower levels than those of the other treatment groups at all times; however, significant differences were only observed at 6 h, where the HDPB group (6.419 ± 4.257 μM) had significantly lower acetaldehyde levels compared to the PLA group (7.477 ± 4.313 μM) (*p* = 0.0251). This is consistent with findings from medical studies highlighting the role of acetaldehyde in alcohol metabolism and its potential impact on hangover severity [59]. These findings reinforce the potential benefits of using a combination of HD and PL, a natural plant-derived extract, in managing alcohol-related adverse effects.

## 4. Conclusions

This study demonstrated the potential hangover-relieving effects of a group of extracts (HDPB and HDGB) when added to HD in individuals aged 19 to 40 years who had previously experienced hangovers. A double-blind, randomized, PLA-controlled crossover trial showed that blood alcohol levels in the HDPB group were significantly lower than those in the PLA group at 0.5 h after ingestion (*p <* 0.05). At 6 h after ingestion, both the HDB and HDPB groups had significantly lower blood alcohol concentrations than the PLA group (*p* < 0.05). Notably, although statistically significant differences were not observed at all time points, the HDPB group consistently had lower blood alcohol levels than the PLA group throughout this study. Similarly, the results for blood acetaldehyde showed that the HDPB group had significantly lower levels than the PLA group at 6 h after ingestion and maintained consistently lower levels than the PLA group at all time points. These results confirm that the consumption of HDB and HDPB foods can significantly improve hangover symptoms by reducing blood acetaldehyde concentrations and blood alcohol concentrations.

## Figures and Tables

**Table 1:** Timeline for human clinical trials.

Period		Screening ^1^	Active Treatment ^2^							
Visit		1	2		3		4		5	
Day		−14	0	1	0	1	0	1	0	1
Window Period (Day) ^3^					+7		+7		+7	
Written consent		✓								
Demographics ^4^		✓								
Lifestyle research ^5^		✓								
Medical and surgical history ^6^		✓	✓							
Medication history, non-medication history ^7^		✓	✓	✓	✓	✓	✓	✓	✓	✓
Physical examination		✓	✓	✓	✓	✓	✓	✓	✓	✓
Vital signs (blood pressure, pulse) ^8^		✓	✓	✓	✓	✓	✓	✓	✓	✓
Body instrumentation ^9^	Height and BMI	✓								
	Weight	✓	✓	✓		✓		✓		✓
Clinical pathology ^10^		✓		✓		✓		✓		✓
Pregnancy reaction test ^10^		✓								
Alcohol degradation genetic testing ^11^		✓								
Breath alcohol test ^12^			✓		✓		✓		✓	
Drinking habits survey		✓	✓		✓		✓		✓	
Validity evaluation	Blood Alcohol, acetaldehyde concentrations ^13^		✓	✓	✓	✓	✓	✓	✓	✓
	Alcohol Hangover Scale (AHS) ^14^		✓	✓	✓	✓	✓	✓	✓	✓
Evaluating human subject suitability		✓	✓							
Randomization			✓							
Consumption of human investigational foods/alcohol ^15^			✓		✓		✓		✓	
Checking for adverse events			✓	✓	✓	✓	✓	✓	✓	✓
Human subject training ^16^		✓		✓		✓		✓		

^1^ Visit 2 (Day 0) occurred within 14 days of Visit 1. Visit 1 and Visit 2 (Day 0) occurred on the same day. If some tests were missing from Visit 1, they could be performed before randomization at Visit 2 (Day 0). ^2^ Visits 2, 3, 4, and 5 took place overnight at the site and included the same diet 2 h before ingestion of the investigational food. ^3^ Visits 3, 4, and 5 (Day 0) were performed after a rest period of 7 days after the fallow period. ^4^ At Visit 1, gender, date of birth, and age were asked about. ^5^ At Visit 1, smoking, exercise, and total sleep duration were asked about. ^6^ Medical history, including surgical procedures within 1 month (30 days) of Visit 1 [however, in the case of gastrointestinal diseases (Crohn’s disease) or gastrointestinal surgery (but not simple appendectomy or hernia surgery), a full medical history was taken with no time limit]. ^7^ A review of the medication and non-medication history within one month (30 days) preceding Visit 1 was conducted. Subsequently, at each visit, an examination of alterations in the medication and non-medication history in comparison to those identified at Visit 1 was undertaken, and the findings were duly recorded. ^8^ At Visits 2, 3, 4, and 5 (Days 0 and 1), vital signs (blood pressure and pulse) were measured 2 h before ingestion of the investigational food and 15 h after completion of drinking. ^9^ Height was measured to the nearest 0.1 cm, and weight was rounded to the nearest 0.1 kg. ^10^ Human clinical trial subjects fasted for 8 h before blood draws and were screened for the following items: Clinical pathology tests at Visit 1 were applicable if results were available within 4 weeks before Visit 1 (excluding pregnancy reactivity tests) and may be re-tested for abnormal results at the discretion of the human clinical trial investigator. Clinicopathologic examinations at Visits 2, 3, 4, and 5 (Day 1) were performed 15 h after completion of drinking. Specimens were stored and analyzed by the external laboratory manual and discarded immediately after analysis without further use. Hematologic tests: WBC, RBC, Hb, Hct, platelet, neutrophil, lymphocyte, monocyte, eosinophil, basophil. Blood chemistry tests: AST (GOT), ALT (GPT), γ-GTP, total protein, blood urea nitrogen (BUN), creatinine, uric acid, alkaline phosphatase (ALP), bilirubin, glucose, total cholesterol, HDL-cholesterol, LDL-cholesterol, triglyceride. Urinalysis: glucose, ketone, bilirubin, RBC (erythrocyte), urobilinogen, Nitrite, WBC (leukocyte). ^11^ Specimens were stored and analyzed by the Exotic Petrographic Institution Manual, discarded immediately after completion of the analysis, and not used for any secondary purposes. ^12^ Before randomization at Visit 2 (Day 0) and before the start of the human clinical trial at Visits 3, 4, and 5 (Day 0) to determine alcohol consumption. ^13^ Samples were collected at Visits 2, 3, 4, and 5 (Days 0 and 1) before drinking and at 0, 0.25, 0.5, 1, 2, 4, 6, and 15 h after completion of drinking. Specimens were stored and analyzed according to the external laboratory manual and discarded immediately after analysis without further use for secondary purposes. ^14^ Visits 2, 3, 4, and 5 (Days 0 and 1) were conducted 1, 2, 4, and 15 h after completion of drinking. ^15^ The human food was consumed in divided portions at 30-minute intervals before alcohol consumption, followed by human food and alcohol (0.9 g/kg body weight) together in divided portions at 30-minute intervals. Fasting was observed after alcohol consumption. ^16^ Human subjects were instructed to abstain from alcohol within 24 h of the next visit at Visit 1 and Visits 2, 3, and 4 (Day 1).

**Table 2:** Ingredients of the PLA group and three experimental beverage groups.

Ingredients (g)	Beverage Group Contents (%)			
	HDB	HDPB	HDGB	PLA
HD ^1^	0.475	0.475	0.475	0
PL ^2^	0	0.1	0	0
GY ^3^	0	0	0.02	0
Sodium bicarbonate	0.023	0.023	0.023	0
Vitamin C	0.020	0.020	0.020	0
Glycine	0.010	0.010	0.010	0
Flavors	0.105	0.105	0.105	0.070
Caramel pigment powder	0	0	0	0.012
Purified water	99.367	99.267	99.347	99.918

Abbreviations: HDB—HD beverage; HDPB—HD beverage combined with 0.1% PL; HDGB—HD beverage combined with 0.02% GY; PLA—placebo; HD*—H. dulcis* extract; PL—*P. lobata* extract; GY—glutathione yeast extract. ^1^ Concentrated aqueous HD: 60 Brix, 50% solid on drying. ^2^ Concentrated aqueous PL: 50 Brix, 40% solid on drying. ^3^ Powder GY: yeast extract 100% (*L*-glutathione > 10%).

**Table 3:** Demographic information and pre-intake characteristics of human subjects (PP set).

Variables		G1 (*n =* 4)	G2 (*n =* 5)	G3 (*n =* 3)	G4 (*n =* 4)	G5 (*n =* 4)	G6 (*n =* 4)	*p*-Value ^1^
Gender *n* (%)	Male	3 (75.00)	3 (60.00)	1 (33.33)	2 (50.00)	2 (50.00)	1 (25.00)	0.8946 (F)
	Female	1 (25.00)	2 (40.00)	2 (66.67)	2 (50.00)	2 (50.00)	3 (75.00)	
Age	Mean ± SD	30.50 ± 5.26	32.80 ± 4.38	26.33 ± 1.53	28.25 ± 3.30	28.25 ± 6.55	29.00 ± 2.16	0.2075 (K)
	Min, Max	26.00, 38.00	28.00, 40.00	25.00, 28.00	24.00, 32.00	24.00, 38.00	27.00, 32.00	
Whether you smoke, *n* (%)	Yes	1 (25.00)	2 (40.00)	1 (33.33)	1 (25.00)	1 (25.00)	0 (0.00)	0.9525 (F)
	No	3 (75.00)	3 (60.00)	2 (66.67)	3 (75.00)	3 (75.00)	4 (100.00)	
Smoking amount (cigarette/day)	Mean ± SD	10.00	6.50 ± 4.95	7.00	1.00	5.00	NS ^2^	0.5252(K)
	Min, Max	10.00	3.00, 10.00	7.00	1.00	5.00	NS	
Exercise or not *n* (%)	No	1 (25.00)	2 (40.00)	0 (0.00)	1 (25.00)	1 (25.00)	0 (0.00)	0.7085 (F)
	1–2 times/week	3 (75.00)	1 (20.00)	1 (33.33)	1 (25.00)	1 (25.00)	3 (75.00)	
	3–4 times/week	0 (0.00)	2 (40.00)	2 (66.67)	2 (50.00)	1 (25.00)	1 (25.00)	
	5–6 times/week	0 (0.00)	0 (0.00)	0 (0.00)	0 (0.00)	1 (25.00)	0 (0.00)	
	Daily	0 (0.00)	0 (0.00)	0 (0.00)	0 (0.00)	0 (0.00)	0 (0.00)	
Total sleep time (h/day)	Mean ± SD	7.75 ± 0.50	7.60 ± 0.89	6.67 ± 0.58	7.25 ± 0.96	7.50 ± 0.58	7.50 ± 1.00	0.4311(K)
	Min, Max	7.00, 8.00	6.00, 8.00	6.00, 7.00	6.00, 8.00	7.00, 8.00	6.00, 8.00	
Height (cm)	Mean ± SD	174.03 ± 11.11	169.46 ± 10.83	167.43 ± 1.59	172.33 ± 9.24	175.33 ± 10.56	166.63 ± 3.20	0.8130 (K)
	Min, Max	158.30, 184.30	157.30, 183.80	165.60, 168.50	164.20, 182.50	164.20, 184.60	164.70, 171.40	
Weight (kg)	Mean ± SD Min, Max	66.48 ± 13.79	64.26 ± 11.28	55.77 ± 1.66	68.13 ± 12.18	70.38 ± 12.53	63.10 ± 6.25	0.6024 (A)
		48.00, 78.60	50.30, 76.60	54.20, 57.50	57.60, 81.50	57.60, 83.90	57.20, 69.50	

^1^ *p-*value for Chi-square test (C) or Fisher’s exact test (F) in categorical variables, *p-*value for ANOVA (A) or Kruskal–Wallis test (K) in continuous variables. ^2^ NS, non-smoker.

**Table 4:** Hematology and blood biochemical tests at 15 h post-consumption for safety evaluation (safety set).

Parameters ^1^	HDB (*n* = 27)	HDPB (*n* = 28)	HDGB (*n = 2*7)	PLA (*n = 2*7)	*p-*Value ^2^
WBC (10^3^/μL)	6.2 ± 1.7	6.0 ± 1.4	6.6 ± 1.8	6.3 ± 1.7	0.7388 (K)
RBC (10^6^/μL)	4.63 ± 0.46	4.6 ± 0.48	4.61 ± 0.49	4.62 ± 0.47	0.9941 (A)
Hb (g/dL)	14.0 ± 1.4	14.0 ± 1.5	14.0 ± 1.5	14.0 ± 1.4	1.0000 (A)
Hct (%)	43.5 ± 4.1	43 ± 4.1	43.5 ± 4.3	43.2 ± 4.0	0.9962 (A)
Platelet (10^3^/μL)	277 ± 64	262 ± 54	270 ± 63	274 ± 56	0.8224 (A)
Neutrophil (%)	45.7 ± 8.4	48 ± 8.3	47.9 ± 9.4	45.3 ± 8.4	0.6103 (A)
Lymphocyte (%)	42.8 ± 8.7	41.1 ± 9.1	41.3 ± 9.5	42.9 ± 9.0	0.8278 (A)
Monocyte (%)	7.1 ± 1.7	7.2 ± 1.7	6.6 ± 1.2	7.3 ± 1.3	0.2959 (K)
Eosinophil (%)	3.8 ± 1.9	3.5 ± 1.6	3.5 ± 2.2	3.8 ± 2.3	0.9384 (K)
Basophil (%)	0.64 ± 0.34	0.73 ± 0.30	0.68 ± 0.34	0.63 ± 0.31	0.6845 (A)
AST (GOT) (U/L)	19.4 ± 7.3	18.8 ± 5.9	19.2 ± 7.2	17.3 ± 4.8	0.6886 (K)
ALT (GPT) (U/L)	19 ± 15	17 ± 12	15 ± 11	15 ± 11	0.8195 (K)
γ-GTP (U/L)	23 ± 23	21 ± 18	21 ± 17	23 ± 24	0.9917 (K)
Total protein (g/dL)	7.4 ± 0.39	7.16 ± 0.29	7.22 ± 0.33	7.22 ± 0.23	0.9486 (K)
BUN (mg/dL)	12.9 ± 2.7	12.8 ± 3.1	13.4 ± 3.3	13.2 ± 2.4	0.9703 (K)
Creatinine (mg/dL)	0.81 ± 0.15	0.80 ± 0.16	0.81 ± 0.14	0.78 ± 0.16	0.8200 (A)
Uric acid (mg/dL)	5.8 ± 1.1	6.0 ± 1.3	5.9 ± 1.3	5.7 ± 1.3	0.7620 (A)
ALP (U/L)	62 ± 22	61 ± 25	62 ± 22	61 ± 22	0.9848 (K)
Bilirubin (mg/dL)	0.77 ± 0.32	0.72 ± 0.24	0.78 ± 0.29	0.77 ± 0.29	0.9394 (K)
Glucose (mg/dL)	74 ± 5.4	76.2 ± 6.6	74.6 ± 5.0	74.2 ± 5.7	0.4531 (A)
Total cholesterol (mg/dL)	201 ± 38	197 ± 44	193 ± 46	201 ± 42	0.5444 (K)
HDL cholesterol (mg/dL)	65 ± 14	64 ± 12	65 ± 14	67 ± 14	0.8874 (A)
LDL cholesterol (mg/dL)	117 ± 34	113 ± 42	111 ± 43	117 ± 41	0.6057 (K)
Triglyceride (mg/dL)	104 ± 37	113 ± 46	104 ± 41	106 ± 36	0.8643 (K)

Values are presented as mean ± SD. ^1^ Hematologic tests: white blood cell (WBC), red blood cell (RBC), hemoglobin (Hb), hematocrit (Hct), platelet, neutrophil, lymphocyte, monocyte, eosinophil, basophil. Blood chemistry tests: AST (GOT), ALT (GPT), γ-GTP, total protein, blood urea nitrogen (BUN), creatinine, uric acid, alkaline phosphatase (ALP), bilirubin, glucose, total cholesterol, HDL-cholesterol, LDL-cholesterol, triglyceride. ^2^ *p-*value for ANOVA (A) or Kruskal–Wallis test (K).

**Table 5:** Vital signs (blood pressure and pulse) (safety set).

Parameters		HDB (*n =* 27)	HDPB (*n = 2*8)	HDGB (*n = 2*7)	PLA (*n =* 27)	*p*-Value ^1^
Systolic blood pressure (mmHg)	Baseline (before ingestion)	116 ± 13	115 ± 11	116 ± 13	117 ± 13	0.9602 (A)
	15 h after drinking	112 ± 12	111 ± 12	114 ± 11	114 ± 12	
	Change from baseline	−4.0 ± 6.9	−4.0 ± 9.3	−1.7 ± 8.7	−2.8 ± 10	0.4753 (K)
	*p*-value ^2^	0.0060 **	0.0309 *	0.3416	0.1709	
Diastolic blood pressure (mmHg)	Baseline (before ingestion)	71 ± 14	71 ± 13	70 ± 11	73 ± 13	0.8774 (K)
	15 h after drinking	69 ± 12	66 ± 12	69 ± 11	69 ± 11	
	Change from baseline	−2.6 ± 12.4	−5.4 ± 11.7	−0.15 ± 9.12	−4.0 ± 10.0	0.3476 (A)
	*p*-value ^2^	0.2807	0.0227 *	0.9321	0.0462 *	
Pulse (Times/min)	Baseline (before ingestion)	82 ± 12	83 ± 12	83 ± 11	82 ± 10	0.9688 (A)
	15 h after drinking	83 ± 14	80 ± 12	83 ± 15	85 ± 13	
	Change from baseline	1.3 ± 13.4	−2.0 ± 12.8	−0.04 ± 10.84	3.2 ± 9.3	0.4186 (A)
	*p*-value ^2^	0.6299	0.4082	0.9857	0.0888	

Values are presented as mean ± SD. ^1^ *p*-value for ANOVA (A) or Kruskal–Wallis test (K). ^2^ *p*-value for the paired *t*-test as * *p* < 0.05 and ** *p* < 0.01 versus the PLA.

**Table 6:** Hourly AHS (points) from the FA and PP sets.

Symptoms	HDB (*n =* 24)	HDPB (*n = 2*4)	HDGB (*n = 2*4)	PLA (*n =* 24)	*p*-Value ^1^
Total score	3.8 ± 3.3	3.6 ± 3.3	3.2 ± 3.0	3.4 ± 2.7	0.3951
Thirst	2.1 ± 2.0	2.2 ± 2.0	2.3 ± 2.0	2.0 ± 1.9	0.7680
Hangover	0.50 ± 0.72	0.54 ± 0.93	0.25 ± 0.61	0.25 ± 0.61	0.1147
Fatigue	0.92 ± 1.06	0.63 ± 0.92	0.63 ± 1.17	0.75 ± 1.03	0.5307
Headache	0.08 ± 0.28	0.17 ± 0.64	0.04 ± 0.20	0.00 ± 0.00	0.4491
Dizziness/Fainting	0.04 ± 0.20	0.00 ± 0.00	0.00 ± 0.00	0.00 ± 0.00	0.3982
Anorexia	0.00 ± 0.00	0.00 ± 0.00	0.00 ± 0.00	0.00 ± 0.00	NA ^2^
Gastrointestinal disorder	0.08 ± 0.28	0.04 ± 0.20	0.00 ± 0.00	0.04 ± 0.20	0.2645
Nausea	0.04 ± 0.20	0.00 ± 0.00	0.00 ± 0.00	0.00 ± 0.00	0.3982
Heart palpitations	0.04 ± 0.20	0.00 ± 0.00	0.00 ± 0.00	0.00 ± 0.00	0.3982

Values are presented as mean ± SD. ^1^ Compared within groups; *p*-value for RM ANOVA. ^2^ NA, not applicable.

**Table 7:** Variations in blood alcohol and acetaldehyde concentrations by group from 0 to 15 h after drinking (PP set).

Variables	Concentration ^1^	HDB (*n =* 24)	HDPB (*n = 2*4)	HDGB (*n = 2*4)	PLA (*n =* 24)	*p-*Value ^2^
Blood Alcohol (%)	AUC	0.64 ± 0.22	0.62 ± 0.25	0.67 ± 0.26	0.66 ± 0.24	0.0800
	C_max_	0.113 ± 0.034	0.109 ± 0.036	0.122 ± 0.050	0.112 ± 0.035	0.2263
	T_max_	1.09 ± 0.58	1.01 ± 0.57	1.10 ± 0.87	1.01 ± 0.51	0.7620
Blood Acetaldehyde (µM)	AUC	119 ± 63	109 ± 60	118 ± 66	121 ± 63	0.1000
	C_max_	14.0 ± 6.0	13.1 ± 6.2	14.8 ± 7.4	14.0 ± 6.5	0.4342
	T_max_	0.69 ± 0.51	1.0 ± 1.4	1.1 ± 1.2	1.3 ± 3.0	0.5489

Values are presented as mean ± SD. ^1^ AUC, area under the concentration–time curve; C_max_, peak blood alcohol concentration; T_max_, time to reach C_max_. ^2^ *p*-value for the paired *t*-test (compared between groups).

## Data Availability

The original contributions presented in this study are included in the article; further inquiries can be directed to the corresponding author.
